# Supplementary material for: Cost-utility analysis of de-escalating biological disease-modifying anti-rheumatic drugs in patients with rheumatoid arthritis
Source: PLoS One. 2020 Jan 2;15(1):e0226754. doi: 10.1371/journal.pone.0226754 (PMC6939943; doi:10.1371/journal.pone.0226754)
Supplement: S1 Table — (DOCX) [file pone.0226754.s001.docx]

**S1 Table. Calculation of pharmaceutical costs.**

| **Trade name** | **Class** | **Substance** | **Biosimilar** | **mg per  package** | **Pharmacy  retail price** | **Manufacturer price** | **Manufacturer discount** | **Pharmacy discount** | **Co- payment** | **Costs  per package** | **Costs  per mg** | **Dosing recommendation** | **mg  per year** | **Costs  per half-year** | **Source** |
| --- | --- | --- | --- | --- | --- | --- | --- | --- | --- | --- | --- | --- | --- | --- | --- |
| Enbrel | bDMARD | Etanercept | no | 600 | 5,231.36 | 4,221.30 | 295.49 | 1.77 | 10.00 | 4,924.10 | 8.21 | 50 mg per week | 2,600 | 10,668.88 | [1] |
| Benepali | bDMARD | Etanercept | yes | 600 | 4,231.35 | 3,405.43 | 238.38 | 1.77 | 10.00 | 3,981.20 | 6.64 | 50 mg per week | 2,600 | 8,625.93 | [1] |
| Erelzi | bDMARD | Etanercept | yes | 600 | 4,231.35 | 3,405.43 | 238.38 | 1.77 | 10.00 | 3,981.20 | 6.64 | 50 mg per week | 2,600 | 8,625.93 | [1] |
| Humira | bDMARD | Adalimumab | no | 240 | 5,231.36 | 4,221.30 | 295.49 | 1.77 | 10.00 | 4,924.10 | 20.52 | 40 mg every 2 weeks | 1,040 | 10,668.88 | [1] |
| Remicade | bDMARD | Infliximab | no | 500 | 4,674.97 | 3,767.36 | 263.72 | 1.77 | 10.00 | 4,399.48 | 8.80 | 3 mg per kg every 8 weeks:  0.75*68.4kg*3mg +  0.25*84.3kg*3mg = 217 mg | 1,411 | 6,205.47 | [1] |
| Flixabi | bDMARD | Infliximab | yes | 500 | 4,157.29 | 3,345.01 | 234.15 | 1.77 | 10.00 | 3,911.37 | 7.82 | 3 mg per kg every 8 weeks:  0.75*68.4kg*3mg +  0.25*84.3kg*3mg = 217 mg | 1,411 | 5,516.99 | [1] |
| Inflectra | bDMARD | Infliximab | yes | 500 | 3,506.19 | 2,813.80 | 196.97 | 1.77 | 10.00 | 3,297.45 | 6.59 | 3 mg per kg every 8 weeks:  0.75*68.4kg*3mg +  0.25*84.3kg*3mg = 217 mg | 1,411 | 4,651.06 | [1] |
| Remsima | bDMARD | Infliximab | yes | 500 | 3,752.81 | 3,015.01 | 211.05 | 1.77 | 10.00 | 3,529.99 | 7.06 | 3 mg per kg every 8 weeks:  0.75*68.4kg*3mg +  0.25*84.3kg*3mg = 217 mg | 1,411 | 4,979.05 | [1] |
| Kineret | bDMARD | Anakinra | no | 2,800 | 1,053.75 | 824.77 | 57.73 | 1.77 | 10.00 | 984.25 | 0.35 | 100 mg per day | 36,500 | 6,415.18 | [2] |
| Cimzia | bDMARD | Certolizumab pegol | no | 1,200 | 4,610.45 | 3,714.72 | 260.03 | 1.77 | 10.00 | 4,338.65 | 3.62 | 200 mg every 2 weeks | 5,200 | 9,400.41 | [1] |
| Simponi | bDMARD | Golimumab | no | 150 | 5,308.97 | 4,284.62 | 299.92 | 1.77 | 10.00 | 4,997.28 | 33.32 | 50 mg per month | 600 | 9,994.55 | [2] |
| MabThera | bDMARD | Rituximab | no | 500 | 2,044.49 | 1,621.26 | 113.49 | 1.77 | 10.00 | 1,919.23 | 3.84 | 1000 mg per 6 month | 2,000 | 3,838.46 | [1] |
| Rixathon | bDMARD | Rituximab | yes | 1,000 | 3,639.19 | 2,922.31 | 204.56 | 1.77 | 10.00 | 3,422.86 | 3.42 | 1000 mg per 6 month | 2,000 | 3,422.86 | [1] |
| Truxima | bDMARD | Rituximab | yes | 500 | 1,644.48 | 1,294.90 | 90.64 | 1.77 | 10.00 | 1,542.07 | 3.08 | 1000 mg per 6 month | 2,000 | 3,084.13 | [1] |
| Orencia | bDMARD | Abatacept | no | 750 | 1,671.12 | 1,316.64 | 92.16 | 1.77 | 10.00 | 1,567.19 | 2.09 | 750 mg per month | 9,000 | 9,403.11 | [2] |
| Kevzara | bDMARD | Sarilumab | no | 1,200 | 4,843.68 | 3,905.00 | 273.35 | 1.77 | 10.00 | 4,558.56 | 3.80 | 200 mg every 2 weeks | 5,200 | 9,876.88 | [2] |
| RoActemra | bDMARD | Tocilizumab | no | 1,944 | 5,231.36 | 4,221.30 | 295.49 | 1.77 | 10.00 | 4,924.10 | 2.53 | 162.6 mg per week | 8,455 | 10,708.40 | [1] |
| Methotrexat AL | csDMARD | Methotrexate | no | 300 | 304.24 | 231.95 | 17.63 | 1.77 | 10.00 | 274.84 | 0.92 | 25 mg per week | 1,300 | 595.49 | [1] |
| Leflunomid | csDMARD | leflunomide | no | 2,000 | 280.32 | 213.03 | 0.00 | 1.77 | 10.00 | 268.55 | 0.13 | 20mg per day | 7,300 | 490.10 | [1] |
| Prednisolon Galen | other | Prednisolon | no | 1,000 | 16.78 | 4.58 | 0.00 | 1.77 | 5.00 | 10.01 | 0.01 | 10 mg per day | 3,650 | 18.27 | [3] |
| Ibuprofen AbZ | other | Ibuprofen | no | 80,000 | 16.97 | 4.73 | 0.00 | 1.77 | 5.00 | 10.20 | 0.00 | 1200 per day | 438,000 | 27.92 | [4] |
| Diclofenac AbZ | other | Diclofenac | no | 5,000 | 14.32 | 2.64 | 0.00 | 1.77 | 5.00 | 7.55 | 0.00 | 75 mg per day | 27,375 | 20.67 | [4] |

We considered two categories of direct costs in our model: pharmaceutical costs and all other direct costs. Pharmaceutical costs consisted of the average costs of (a) one csDMARD, (b) one bDMARD and (c) concomitant treatment with one NSAID and one glucocorticoid. We calculated the average costs, to the payer, per milligram (mg) of the largest pack size available for each substance.

First, we subtracted the manufacturer discount (7%), pharmacy discount (€1.77 per package) and patient co-payment (10% of retail price, min. €5, max. €10) from the pharmacy retail price for 2017. Second, we divided the calculated costs per package by the amount of milligrams per package. The bDMARDs we considered were as follows: etanercept and two biosimilars (€8.21/€6.64/€6.64 per mg), adalimumab (€20.52 per mg), infliximab and three biosimilars (€8.80/€7.82/€7.06/€6.59 per mg), anakinra (€0.35 per mg), certolizumab pegol (€3.62 per mg), golimumab (€33.32 per mg), rituximab and two biosimilars (€3.84/€3.42/€3.08 per mg), abatacept (€2.09 per mg), sarilumab (€3.80 per mg) and tocilizumab (€2.53 per mg). The csDMARDs we considered were methotrexate (€0.92 per mg) and leflunomide (€0.13 per mg). Concomitant medication comprised prednisolone (€0.01 per mg) and the NSAIDs ibuprofen and diclofenac (less than €0.01 per mg). Third, to calculate the required dose in mg per cycle, we multiplied prices per mg by recommended doses from product information provided by the European Medicines Agency (EMA) [2], recommendations of the German Society of Rheumatology (Deutsche Gesellschaft für Rheumatologie) [3] and the German League Against Rheumatism (Deutsche Rheumaliga), [4] and published literature [1]. Lastly, we calculated average costs in each category for a six-month cycle, which yielded costs of (a) €7,416 for bDMARDs, (b) €543 for csDMARDS and €22 for concomitant medication (for detailed information, see Appendix table 1).

All other direct costs were taken from Huscher et al. [5], who report average direct costs per disease activity state for patients with RA in Germany in 2011 based on the Health Assessment Questionnaire (HAQ) [6,7]. Non-pharmaceutical costs in our study comprised those of hospitalization, rehabilitation, physician visits, joint replacement surgery, physiotherapy and imaging [5]. To reflect growth in the expenditure of statutory health insurers in Germany from 2011 to 2017, we inflated costs by 29.6% [8]. Thus, all other direct costs for patients aged ≤ 64 years amounted to €747 (€1,1012 for > 65 years) when in REM, €1,158 (€1,1210) when in LDA and €1,856 (€1,941) when in M/HDA.

Total direct costs per state per patient aged ≤ 64 years were €8,729 (€8,994 for > 65 years) for REM, €9,140 (€9,192) for LDA and €9,838 (€9,922) for M/HDA under standard care. For tapering, we considered only 50% of the bDMARD costs, yielding total direct costs of €5,021 (€5,286) for TP_REM and €5,432 (€5,484) for TP_LDA. For withdrawal, we calculated costs of €1,313 (€1,577) for patients in WD-REM and €1,723 (€1,776) patients in WD-LDA.

**Literature**

1. Smolen JS, Aletaha D, McInnes IB. Rheumatoid arthritis. The Lancet. 2016;388: 2023–2038. doi:10.1016/S0140-6736(16)30173-8

2. European Medicince Agency (EMA). European Medicines Agency - Find medicine - European public assessment reports. In: European public assessment reports [Internet]. 2018 [cited 14 Mar 2018].

3. Krüger K, Wollenhaupt J, Albrecht K, Alten R, Backhaus M, Baerwald C, et al. S1-Leitlinie der Deutschen Gesellschaft für Rheumatologie: Handlungsempfehlungen der DGRh zur sequenziellen medikamentösen Therapie der rheumatoiden Arthritis 2012: adaptierte EULAR Empfehlungen und aktualisierter Therapiealgorithmus. Dtsch Ges Für Rheumatol. 2012.

4. DRL BV. NSAR | Deutsche Rheuma-Liga Bundesverband e.V. In: Nicht-steroidale Antirheumatika [Internet]. 2017 [cited 13 Jul 2017].

5. Huscher D, Mittendorf T, von Hinüber U, Kötter I, Hoese G, Pfäfflin A, et al. Evolution of cost structures in rheumatoid arthritis over the past decade. Ann Rheum Dis. 2015;74: 738–745. doi:10.1136/annrheumdis-2013-204311

6. Fries JF, Spitz P, Kraines RG, Holman HR. Measurement of patient outcome in arthritis. Arthritis Rheum. 1980;23: 137–145.

7. Bruce B, Fries JF. The Stanford Health Assessment Questionnaire: Dimensions and Practical Applications. Health Qual Life Outcomes. 2003;1: 20. doi:10.1186/1477-7525-1-20

8. Statistisches Bundesamt (Destatis). Staat & Gesellschaft - Gesundheitsausgaben. In: Gesundheitsausgaben in Deutschland [Internet]. 2018 [cited 9 Aug 2018].
