# Supplementary material for: Cost-utility analysis of de-escalating biological disease-modifying anti-rheumatic drugs in patients with rheumatoid arthritis
Source: PLoS One. 2020 Jan 2;15(1):e0226754. doi: 10.1371/journal.pone.0226754 (PMC6939943; doi:10.1371/journal.pone.0226754)
Supplement: S2 Table — (DOCX) [file pone.0226754.s002.docx]

**S2 Table. Detailed overview of study pool for estimation of transition probabilities.**

| **Author** | **Year** | **Journal** | **Country** | **Name** | **Sub-stance** | **Design** | **SC/**  **TP/**  **WD** | **Intervention** | **IG/**  **CG** | **Definition of REM** | **DAS28** | **SD** | **ESR/CRP** | **N** | **Model State** | |
| --- | --- | --- | --- | --- | --- | --- | --- | --- | --- | --- | --- | --- | --- | --- | --- | --- |
| Kremer | 2005 | Arthritis Rheumatol | . | . | ABA | RCT | SC | ABA 10 mg + MTX | IG | DAS28 < 2.6 | 5,5 | 0,63 | CRP | 115 | M/HDA | |
| Westhovens et al. | 2006 | Arthritis Rheumatol | FI | ATTRACT | INF | RCT | SC | INF 3mg + MTX and INF 10mg + MTX | IG | DAS28 < 2.6 | 5,7^a^ |  |  | 721 | M/HDA | |
| Emery et al. | 2008 | Lancet | EU, LA, AS, AU | COMET | ETA | RCT | SC | ETA + MTX | IG | DAS28 < 2.6 | 6,5 | 1 |  | 274 | M/HDA | |
| Schiff et al. | 2008 | Ann Rheum Dis | Global | ATTEST | ABA | RCT | SC | INF + MTX | IG | DAS28 < 2.6 | 6,9 | 1 | ESR | 156 | M/HDA | |
| Schiff et al. | 2008 | Ann Rheum Dis | Global | ATTEST | INF | RCT | SC | ABA + MTX | IG | DAS28 < 2.6 | 6,8 | 0,9 | ESR | 165 | M/HDA | |
| Ruubert-Roth | 2010 | Rheumatology | Global | MIRROR | RITU | RCT | SC | RTX + MTX | IG | DAS28 < 2.6 | 6.7 | 1.0 | ESR | 378 | M/HDA | |
| Tanaka et al. | 2010 | Ann Rheum Dis | JP | RRR | INF | SAT | WD | MTX mono | IG | DAS28 < 2.6 | < 2.6^b^ |  | ESR | 114 | LDA | |
| Kim | 2012 | Int J Rheum Dis | APR | APPEAL | ETA | RCT | SC | ETA + MTX | IG | DAS28 < 2.6 | 6.1 | 1.1 | ESR | 197 | M/HDA | |
| Kavanaugh | 2013 | Ann Rheum Dis | NA, SA, EU, AF,  NZ, AU | OPTIMA | ADA | RCT | SC | ADA + MTX | IG | . | 6 | 1 | CRP | 515 | M/HDA | |
| Smolen et al. A | 2013 | Lancet | EU, LA, AS, AU | PRESERVE | ETA | RCT | SC | ETN 50mg/wk + MTX | CG | DAS28 < 2.6 | 4,4 | 0,4 | ESR | 834 | M/HDA | |
| Smolen et al. B | 2013 | Lancet | EU, LA, AS, AU | PRESERVE | ETA | RCT | SC | ETN 50mg/wk + MTX | CG | DAS28 < 2.6 | 2 | 0,6 | ESR | 202 | LDA | |
| Smolen et al. C | 2013 | Lancet | EU, LA, AS, AU | PRESERVE | ETA | RCT | TP | ETN 25mg/wk | IG | DAS28 < 2.6 | 2,1 | 0,6 | ESR | 202 | LDA | |
| Smolen et al. D | 2013 | Lancet | EU, LA, AS, AU | PRESERVE | ETA | RCT | WD | PBO + MTX | IG | DAS28 < 2.6 | 2,1 | 0,6 | ESR | 200 | LDA | |
| Yoo et al. A | 2013 | Ann Rheum Dis | EU, AS,  LA, ME | PLANETRA | INF | RCT | SC | Biosimilar + MTX | IG | . | 5,9 | 0,8 | CRP | 245 | M/HDA | |
| Yoo et al. B | 2013 | Ann Rheum Dis | EU, AS,  LA,  ME | PLANETRA | INF | RCT | SC | INF + MTX | CG | . | 5,8 | 0,9 | CRP | 251 | M/HDA | |
| Dougados | 2014 | Ann Rheum Dis | NL | ACT-RAY | TOC | RCT | SC | TCZ + MTX | IG | DAS28 < 2.6 | 6,3 | 1 | ESR | 277 | HDA | |
| Emery et al. A | 2014 | New Engl J Med | EU, AS | PRIZE | ETA | RCT | WD | MTX + PBO | IG | DAS28 < 2.6 | < 2.6^b^ |  |  | 65 | REM | |
| Emery et al. B | 2014 | New Engl J Med | EU, AS | PRIZE | ETA | RCT | SC | ETA + MTX | CG | DAS28 < 2.6 | < 2.6^b^ |  |  | 63 | REM | |
| Horslev-Petersen | 2014 | Ann Rheum Dis | DK | OPERA | ADA | RCT | SC | ADA + MTX | IG | DAS28 < 2.6 | 5,5 |  | CRP | 89 | M/HDA | |
| Machado et al. | 2014 | JCR-J Clin Rheumatol | LA | . | ETA | RCT | SC | ETA + MTX | IG | DAS28 < 2.6 | 6,6 | 0,7 | ESR | 281 | HDA | |
| Nam et al. A | 2014 | Ann Rheum Dis | UK | IDEA | INF | RCT | SC | MTX + IFX | CG | DAS28 < 2.6 | 5,3 | 2,1 |  | 55 | M/HDA | |
| Nam et al. B | 2014 | Ann Rheum Dis | UK | EMPIRE | ETA | RCT | WD | MTX + PBO | IG | . | 2,6 |  | CRP | 41 | REM | |
| Schiff et al. A | 2014 | Ann Rheum Dis | NA, SA | AMPLE | ABA, ADA | RCT | SC | ABA + MTX | IG | . | 5,5 | 1,1 | CRP | 318 | M/HDA | |
| Schiff et al. B | 2014 | Ann Rheum Dis | NA, SA | AMPLE | ABA, ADA | RCT | SC | ADA + MTX | CG | . | 5,5 | 1,1 | CRP | 328 | M/HDA | |
| Smolen et al. A | 2014 | Lancet | EU, NA, SA, AF,  AU, NZ | OPTIMA | ADA | RCT | WD | MTX | IG | DAS28 < 2.6 | 2,2 | 0,6 | CRP | 102 | REM or LDA | |
| Smolen et al. B | 2014 | Lancet | EU, NA, SA, AF,  AU, NZ | OPTIMA | ADA | RCT | SC | ADA + MTX | CG | DAS28 < 2.6 | 2 | 0,5 | CRP | 105 | REM or LDA | |
| Tanaka et al. A | 2015 | Ann Rheum Dis | JP | HONOR | ADA | OC | WD | MTX | IG | DAS28 < 2.6 | < 2.6^b^ |  | ESR | 52 | REM | |
| Tanaka et al. B | 2015 | Ann Rheum Dis | JP | HONOR | ADA | OC | SC | ADA + MTX | CG | DAS28 < 2.6 | < 2.6^b^ |  | ESR | 23 | REM | |
| van Herwaarden B | 2015 | BMJ Brit Med J | NL | DRESS | ADA, ETA | RCT | SC | ADA / ETA + MTX | CG | . | 2.5 | 0.8 | ESR | 59 | LDA | |
| Haschka et al. A | 2016 | Ann Rheum Dis | DE | RETRO | TNF | RCT | TP | Half dose of TNF | IG | DAS28 < 2.6 | 1,9 | 0,8 | ESR | 36 | REM | |
| Haschka et al. B | 2016 | Ann Rheum Dis | DE | RETRO | TNF | RCT | WD | Withdrawal of TNF | IG | DAS28 < 2.6 | 2 | 0,8 | ESR | 27 | REM | |
| Haschka et al. C | 2016 | Ann Rheum Dis | DE | RETRO | TNF | RCT | TP | Half dose of TNF | IG | DAS28 < 2.6 | 2 | 0,8 | ESR | 27 | REM | |
| Haschka et al. D | 2016 | Ann Rheum Dis | DE | RETRO | TNF | RCT | SC | Full dose of TNF | CG | DAS28 < 2.6 | 1,8 | 1 | ESR | 38 | REM | |
| Keystone et al. A | 2016 | Rheumatology | CA | CAMEO | ETA | RCT | SC | ETA + MTX | IG | . |  |  | ESR | 47 | REM or LDA | |
| Keystone et al. B | 2016 | Rheumatology | CA | CAMEO | ETA | RCT | SC | ETA + MTX | IG | . |  |  | ESR | 58 | M/HDA | |
|  |  |  |  |  |  |  |  |  |  |  |  |  |  |  |  | |
|  |  |  |  |  |  |  |  |  |  |  |  |  |  |  |  | |
| ABA: abatacept, ADA: adalimumab, ETA: etanercept, INF: infliximab, RITU: rituximab, OC: observational cohort, RCT: randomized controlled trial, SAT: single-arm trial IG: intervention group, CG: control group, SDAIC: simpliﬁed disease activity index criteria, WW: worldwide, AF: Africa, APR: Asia-Pacific Region, AS: Asia AU: Australia, CA: Canada DE: Germany, DK: Denmark, EU: Europe FI: Finland JP: Japan, LA: Latin America, ME: Middle East, NA: North America, NL: Netherlands, NZ: New Zealand, SA: South America, REM: clinical remission LDA: low disease activity, M/HDA: medium or high disease activity, ^a^ calculated based on baseline characteristics, ^b^ not further specified | | | | | | | | | | | | | | | |  |
