# Supplementary material for: Cost-utility analysis of de-escalating biological disease-modifying anti-rheumatic drugs in patients with rheumatoid arthritis
Source: PLoS One. 2020 Jan 2;15(1):e0226754. doi: 10.1371/journal.pone.0226754 (PMC6939943; doi:10.1371/journal.pone.0226754)
Supplement: Attachment — Submitted filename: Review PONE-D-19-15210.docx [file pone.0226754.s007.docx]

This study presents a cost-utility analysis of tapering and/or withdrawing biological agents in patients with rheumatoid arthritis using a Markov model with a time horizon of 30 years. The rationale for this study, as indicated by the authors, is to pool all available evidence from the literature and extrapolate the clinical outcomes to a long-term period. While this sounds reasonable and interesting, there are major issues regarding the model structures and parameter estimation.

Model structures:

In the models for tapering, withdrawal, and tapering followed by withdrawal (Figures 1B, 1C and 1D), the patients in low disease activity (LDA) can go to remission (REM) and vice versus (indicated by the bidirectional arrows). However, the authors made the rules that patients in LDA or REM for at least six months would enter the de-escalation states. Because one model cycle is six months, the patients in LDA can remain in the same state or only go to LDA or REM under tapering, defined by the authors as TP-LDA and TP-REM, respectively. This means that the bidirectional transition between states LDA and REM in the models for de-escalation is inappropriate.

Another concern relates to the model cycle of six months, which is rather long. As indicated in a number of clinical trials (e.g. Haschka et al. Ann Rheum Dis 2016;75:45-51; Moghadam et al. Arthritis Rheum 2016;68:1810-1817), about 10% of patients in remission and under tapering or stopping of bDMARDs entered LDA or had flares after 3 months, which affects costs and health utility. Another possibility is that at month 9, up to 19% and 6% of the patients in remission and under tapering could enter LDA and moderate/high disease activity states, respectively, as indicated in Haschka et al. As the models in review are not able to capture these situations, it is important that the authors examine the impact of ignoring these possible transitions and flares within the 6-month cycles on the model outcomes.

Model parameter estimation:

It is completely unclear how the authors estimate the upper and lower bounds of the model parameters. I have the following questions:

- Which outcomes reported in the included studies did the authors summarize to estimate the pooled probabilities? Were those risk ratios, odds ratios or risk differences? In meta-analysis, both fixed- and random-effects models are used to model effect sizes. I am not aware of any models in meta-analyses that model probability separately in each treatment arm (as an example, the Cochrane Handbook for Systematic Review of Interventions does not mention any methods for summarizing probabilities – see section Types of data and effect measures).
- Assume that there was an appropriate method to summarize probabilities for each treatment arm in the meta-analysis, it would still be inappropriate to use time-independent probabilities for state transitions, as indicated in Table 1 and Table 2 in the manuscript. It is very clear in many studies included in the meta-analysis of the study in review that the probability of transition from remission to low or high disease activity changed over time.
- The authors used an equation, claimed to be standard approach, to estimate transition probabilities per cycle, which I am not aware of (the authors did not provide any reference or proof). As far as I know, a common approach to convert risk over a certain period to risk over a different period is first to convert risk to rate, and then from rate to risk as explained very clearly in several text books and publications for decision analytic modelling and economic evaluations of health care programs (for example, the widely read and highly cited textbook by Andrew Briggs, Mark Sculpher and Karl Claxton: Decision Modelling for Health Economic Evaluation).
- How much clinical heterogeneity was there between studies used for combining the effects? As far as I know, there are substantial differences in the definitions of remission and low disease activity, and in the criteria to start bDMARDs between studies on tapering or stopping bDMARDs. Combining probabilities representing different health status may introduce bias. It should be noted that the Cochrane Handbook for Systematic Review of Interventions recommends not to perform meta-analysis when the heterogeneity is large.
- It is completely clear how the authors estimate the upper and lower bounds of the model parameters for the univariate sensitivity analysis, and the parameters of the distributions of the model parameters for the probabilistic sensitivity analysis. For example, the authors used the Dirichlet distributions of the transition probabilities but did not justify this or explain how the parameters were derived.

Other comments:

- bDMARD in the title should be spelt out
- ICERs should be presented in the Abstract
- Interpretation of the results from the probabilistic sensitivity analysis is not sufficient. The readers would expect to see at which willingness-to-pay thresholds the probabilities of the de-escalation being cost-effective are, for example, 50% and 100%.
- The authors’ claim that their “modelling approach can be transferred easily to different health systems by adapting the cost parameters” does not seem valid. Health utility associated with a health state is most likely different in different countries (for example, see [https://www.ohe.org/publications/can-we-really-compare-and-aggregate-pro-data-between-people-and-settings-implications#](https://www.ohe.org/publications/can-we-really-compare-and-aggregate-pro-data-between-people-and-settings-implications))
