# Supplementary material for: Cost-utility analysis of de-escalating biological disease-modifying anti-rheumatic drugs in patients with rheumatoid arthritis
Source: PLoS One. 2020 Jan 2;15(1):e0226754. doi: 10.1371/journal.pone.0226754 (PMC6939943; doi:10.1371/journal.pone.0226754)
Supplement: Attachment — Submitted filename: Response to Reviewers.docx [file pone.0226754.s008.docx]

# Response to reviewers

Dear reviewers

Thank you very much for the very constructive feedback on our manuscript. We gratefully included your suggestions and adjusted our model structure to improve our analysis. Please find our replies to all your raised comments below.

Kind regards

Benjamin Birkner, Jürgen Rech and Tom Stargardt

# Editor

**Please address all of the comments of the Reviewers. All statistical analyses need to be revised as it has been suggested by Reviewer 1**

Reply:

Thank you very much for the opportunity to improve our manuscript. We addressed all points raised by both reviewers resulting in a change of the model structure (to 3 months), additional sensitivity analyses as well as scenario analyses.

# Reviewer #1

**Model structures:**

## Comment #1

**In the models for tapering, withdrawal, and tapering followed by withdrawal (Figures 1B, 1C and 1D), the patients in low disease activity (LDA) can go to remission (REM) and vice versus (indicated by the bidirectional arrows). However, the authors made the rules that patients in LDA or REM for at least six months would enter the de-escalation states. Because one model cycle is six months, the patients in LDA can remain in the same state or only go to LDA or REM under tapering, defined by the authors as TP-LDA and TP-REM, respectively. This means that the bidirectional transition between states LDA and REM in the models for de-escalation is inappropriate.**

Reply:

Thank you very much for your comments. The rationale for the choice of the bidirectional transition between REM and LDA before tapering/withdrawal was motivated by the de-escalation criteria of stable disease activity for 6 months. However, we understand that you suggest to apply a different model structure where patients who are at least in LDA or REM for 6 months directly progress to our tapering/withdrawal states instead.

We thus made the suggested structural changes to the model and changed the bidirectional relation. Patients in LDA who experience improved disease activity (i.e. REM) now progress to the TP/WD_REM state (see modified model structure in graph after comment 2). This way we still ensure that patients can only be in the de-escalation treatment of stable or improved disease activity.

## Comment #2

**Another concern relates to the model cycle of six months, which is rather long. As indicated in a number of clinical trials (e.g. Haschka et al. Ann Rheum Dis 2016;75:45-51; Moghadam et al. Arthritis Rheum 2016;68:1810-1817), about 10% of patients in remission and under tapering or stopping of bDMARDs entered LDA or had flares after 3 months, which affects costs and health utility. Another possibility is that at month 9, up to 19% and 6% of the patients in remission and under tapering could enter LDA and moderate/high disease activity states, respectively, as indicated in Haschka et al. As the models in review are not able to capture these situations, it is important that the authors examine the impact of ignoring these possible transitions and flares within the 6-month cycles on the model outcomes.**

Reply:

We are very grateful for this comment. We followed your suggestion and believe that the model is now depicting the possible treatment path of patients treated with bDMARDs who are potentially de-escalated more closely.

We revisited all included studies in our study pool and found 3-month results in four RCTs. First, we updated the transition probabilities. Second, we included tunnel states to account for the probability of flares after each cycle of 3 months. We updated all results as well as scenario and sensitivity analyses in the manuscript accordingly. Please see new and old model structure below.

Due to the changes incremental QALYs increased for tapering and decreased for withdrawal and dose reduction followed by withdrawal. Thus, there was a change in magnitude of the ICERs but no change in direction and / or interpretation of study results.

**NEW Model Structure	OLD Model Structure**

REM: Remission; LDA: Low disease activity; M/HDA: Medium to high disease activity; TP_: Tapering; S_: sustained (tunnel state)

**NEW Model Structure	OLD Model Structure**

REM: Remission; LDA: Low disease activity; M/HDA: Medium to high disease activity; WD_: Withdrawal; S_: sustained (tunnel state)

**NEW Model Structure	OLD Model Structure**

REM: Remission; LDA: Low disease activity; M/HDA: Medium to high disease activity; TP/WD_: Tapering/Withdrawal; S_: sustained (tunnel state)

## Comment #3

**Model parameter estimation:**

**It is completely unclear how the authors estimate the upper and lower bounds of the model parameters. I have the following questions:**

**Which outcomes reported in the included studies did the authors summarize to estimate the pooled probabilities? Were those risk ratios, odds ratios or risk differences? In meta-analysis, both fixed- and random-effects models are used to model effect sizes. I am not aware of any models in meta-analyses that model probability separately in each treatment arm (as an example, the Cochrane Handbook for Systematic Review of Interventions does not mention any methods for summarizing probabilities – see section Types of data and effect measures).**

Reply:

Thank you very much for your comment. We think this is a misunderstanding due to us not being clear enough with our approach on the estimation of transitional probabilities. We very much already followed the approach outlined by the reviewer.

We took a three-step approach to estimate transition probabilities:

1. Categorization of studies in pool according to the baseline DAS28 of the study populations
2. Extraction of the number patients in categories REM (DAS28<2.6), LDA (DAS28>2.6 <3.2) and M/HDA(DAS28 >3.2)
3. Calculation of transition probabilities based on cycle length of 3 months using the approach by Briggs et al. The only difference here is that we applied the two steps of transforming to a rate and then to probability in one step (i.e., combined the two formula). [Results are the same compared to the two steps when applying the original formula]

Indeed, we did a random effects pooling but termed it slightly different.

We have rephrased the corresponding paragraph in the manuscript and added corresponding literature (lines 82-104 of the manuscript).

## Comment #4

**Assume that there was an appropriate method to summarize probabilities for each treatment arm in the meta-analysis, it would still be inappropriate to use time-independent probabilities for state transitions, as indicated in Table 1 and Table 2 in the manuscript. It is very clear in many studies included in the meta-analysis of the study in review that the probability of transition from remission to low or high disease activity changed over time.**

Reply:

We agree that computing time dependent transition probabilities would have been the more accurate approach. However, we feel that the duration of included studies in our study pool is too heterogeneous to do so for all parameters. Thus we only implemented (run-)time dependent probabilities for mortality in the original model.

## Comment #5

**The authors used an equation, claimed to be standard approach, to estimate transition probabilities per cycle, which I am not aware of (the authors did not provide any reference or proof). As far as I know, a common approach to convert risk over a certain period to risk over a different period is first to convert risk to rate, and then from rate to risk as explained very clearly in several text books and publications for decision analytic modelling and economic evaluations of health care programs (for example, the widely read and highly cited textbook by Andrew Briggs, Mark Sculpher and Karl Claxton: Decision Modelling for Health Economic Evaluation).**

Reply:

Thank you very much for pointing this out. We apologise for being imprecise. However, we already applied the common approach by Briggs et al. We added the respective citation and rephrased the sentences in line 100-104 to be clearer.

## Comment #6

**How much clinical heterogeneity was there between studies used for combining the effects? As far as I know, there are substantial differences in the definitions of remission and low disease activity, and in the criteria to start bDMARDs between studies on tapering or stopping bDMARDs. Combining probabilities representing different health status may introduce bias. It should be noted that the Cochrane Handbook for Systematic Review of Interventions recommends not to perform meta-analysis when the heterogeneity is large.**

Reply:

Thank you very much. There was no heterogeneity in the definition of remission or low disease activity within our study pool. We used results from RCTs who report the number or share of patients according to DAS28 only. The included studies either defined remission as DAS28 <2.6 and low disease activity as DAS28 <3.2 or reported the numbers of patients for these outcomes solely based on the DAS28 categories of <2.6 and <3.2. We added this remark to our inclusion criteria for our study pool in line 88-89.

## Comment #7

**It is completely clear how the authors estimate the upper and lower bounds of the model parameters for the univariate sensitivity analysis, and the parameters of the distributions of the model parameters for the probabilistic sensitivity analysis. For example, the authors used the Dirichlet distributions of the transition probabilities but did not justify this or explain how the parameters were derived.**

**Reply:**

Thank you very much for this suggestion. We included more details about the origin of upper and lower bounds as well as the chosen distributions for the sensitivity analysis of our model parameters in section 2.3 (beginning from line 148-158).

## Comments #8-11

**Other comments:**

- **bDMARD in the title should be spelt out**
- **ICERs should be presented in the Abstract**
- **Interpretation of the results from the probabilistic sensitivity analysis is not sufficient. The readers would expect to see at which willingness-to-pay thresholds the probabilities of the de-escalation being cost-effective are, for example, 50% and 100%.**
- **The authors’ claim that their “modelling approach can be transferred easily to different health systems by adapting the cost parameters” does not seem valid. Health utility associated with a health state is most likely different in different countries (for example, see** [**https://www.ohe.org/publications/can-we-really-compare-and-aggregate-pro-data-between-people-and-settings-implications#**](https://www.ohe.org/publications/can-we-really-compare-and-aggregate-pro-data-between-people-and-settings-implications)**)**

Reply:

Thank you very much. We revised as follows:

- We spelt out bDMARDs in the title
- ICERs are now added to our abstract
- We added more information on our results of our PSA regarding the interpretation of the CEAC curve and added the thresholds included in our analysis (lines 194-202)
- We understand that our claim is too vague and we added a remark that application to different settings requires adjustment of cost and utility parameters (line 230).

# Reviewer #2

## Comment #1

**This is a new, important and interesting paper. The scientific methodology is valid.
I suggest adding 2 tables for practicing rheumatologist who read the paper: one table that describes all attributes of the studies that were performed on bDMARD de-escalation (setting, number of patients, de-escalation method, result, etc.) and another table on the advantages and disadvantages of bDMARD de-escalation**

Reply:

Thank you very much for providing this feedback. We really appreciate that.

1) Since table 1 is already quite broad, we added another table containing the suggested information to the appendix / supplementary material. Results (i.e. the number of patients in the respective disease states) are not included in this additional table as we show them in detail in table 1.
2) Since the main author of this paper is an economist, we do not feel that we are to judge on the clinical advantages and disadvantages of bDMARD de-escalation. We limit ourselves to providing evidence on the cost-effectiveness of the three approaches.
