# Supplementary material for: Cost-utility analysis of de-escalating biological disease-modifying anti-rheumatic drugs in patients with rheumatoid arthritis
Source: PLoS One. 2020 Jan 2;15(1):e0226754. doi: 10.1371/journal.pone.0226754 (PMC6939943; doi:10.1371/journal.pone.0226754)
Supplement: Attachment — Submitted filename: Response to Reviewers.docx [file pone.0226754.s009.docx]

# Response to reviewers

Dear reviewers

Thank you very much for the very constructive feedback on our manuscript. We rephrased several sentences and included additional information to be clearer to the reader. Please find our replies to all your raised comments below.

Kind regards

Benjamin Birkner, Jürgen Rech and Tom Stargardt

# Editor

**Please address all of the comments of the Reviewers.**

Reply:

Thank you very much for the new opportunity to revise our manuscript. We addressed all comments raised by Reviewer #3 as seen below.

# Reviewer #3

## Comment #1

**Have the authors made all data underlying the findings in their manuscript fully available?

The** [**PLOS Data policy**](http://www.plosone.org/static/policies.action#sharing) **requires authors to make all data underlying the findings described in their manuscript fully available without restriction, with rare exception (please refer to the Data Availability Statement in the manuscript PDF file). The data should be provided as part of the manuscript or its supporting information, or deposited to a public repository. For example, in addition to summary statistics, the data points behind means, medians and variance measures should be available. If there are restrictions on publicly sharing data—e.g. participant privacy or use of data from a third party – those must be specified.**

**Reviewer #3: No**

Reply:

Thank you very much for pointing this out (detailed in comment #4). After crosschecking the reporting of our input parameters as well as our modelling approach, we feel that all the data supplied within our tables and supplementary information together with the model structure would be sufficient to replicate our findings. We will provide the R code for our model to interested readers upon request. We can also supply the code as a supplementary file if this is the common procedure at PLOS One.

## Comment #2

**Page 4, line 46: Should “our starting cohort” be ‘our hypothetical starting cohort’ because all results were based on simulations?**

Reply:

Thank you very much for this advice. We rephrased the sentence to read “our hypothetical starting cohort”.

## Comment #3

**Page 6, lines 81–82: How did the “random effects pooling” fit into the three-step approach mentioned in the next paragraph? If it was part of the three-step approach, I suggest to include the random effects pooling in the three-step approach. If not, I suggest to make the role of random effects pooling more clear and what its relationship with the three-step approach.**

Reply:

We are very grateful for this comment. We added another paragraph detailing the rationale of the random effects pooling to explain our approach more clearly.

## Comment #4

**page 8, Table 1: If I understood the Transition Probabilities section correctly, Table 1 depicted the transition probabilities summarized by the literature review. However, the transition probabilities that were used in the Markov models were estimated from the random effects pooling based on transition probabilities in Table 1. If this is true, I suggest to include the transition probabilities used for the Markov models as Table 1 and the current Table 1 as a supplemental table. Please confirm and clarify in the manuscript.**

Reply:

Thank you very much for your feedback. Your understanding of how we utilized the estimated transition probabilities is correct. We report the transition probabilities among with the other parameters in table 2. However, we feel that table 1 (together with the more detailed explanation of the random effects pooling) greatly adds to the transparency of our approach. Thus we decided to keep table 1 in the manuscript in order to make the process of how we utilized the large body of clinical evidence for the estimation of transition probabilities comprehensible for the reader.

## Comment #5

**Page 12, Table 2: I think “baseline model” was later referred as “base case” or “base case model”. I suggest to use a consistent name throughout the manuscript.**

Reply:

Thank you very much for pointing this out. We changed the title of table 2 and the header of the respective column in table 2 accordingly.

## Comment #6

**Page 16, line 224: The sensitivity analyses could be more informative if the deterministic low-high bounds were more extreme. Taking bDMARDs of pharmaceutical costs under annual costs, the low (8147) and high (21520) bounds were around 15% and 85% of the associated gamma distribution. I thought more extreme values such as 1% and 99% could be useful to provide the lower and upper limits for the results.**

Reply:

Thank you very much for your feedback, we appreciate that. We applied the suggested thresholds of 1% and 99% as additional bounds for bDMARD costs with the effect that incremental costs for the high (low) case increased (decreased). We added another paragraph addressing this issue within section 3.2.
